# Development and usability evaluation of HOPE: A patient-centered mHealth application for HTN self-management in Iran

**DOI:** 10.1371/journal.pone.0344541

**Published:** 2026-06-17

**Authors:** Mohammad Hosein Hayavi-Haghighi, Abdullah Gharibzade, Zahra Mastaneh, Haniyeh Ansarifard

**Affiliations:** 1 Department of Health Information Technology, School of Allied Medical Sciences, Hormozgan University of Medical Sciences, Bandar Abbas, Iran; 2 Department of Cardiology, School of Medicine, Tobacco and Health Research Center, Hormozgan University of Medical Sciences, Bandar Abbas, Iran; 3 Faculty of Para-Medicine, Hormozgan University of Medical Sciences, Bandar Abbas, Iran; Indiana University South Bend, UNITED STATES OF AMERICA

## Abstract

**Background:**

Hypertension (HTN) is a major global health problem and a significant risk factor for cardiovascular disease. Mobile health (mHealth) applications offer an efficient, patient-centered approach to managing chronic conditions like HTN. Given the high prevalence of HTN in Iran, and a recognized lack of approved and scientifically-grounded mHealth applications, this study aimed to address this gap, particularly in Hormozgan Province.

**Objective:**

This study aimed to design and evaluate a HTN self-care application, named HOPE, to facilitate self-management and enable patients to access health services outside of clinical settings.

**Materials and methods:**

The research was conducted in four steps: (1) determination of data elements and functional requirements based on a systematic review of guidelines and feedback from 25 cardiologists and 50 patients using a Likert scale questionnaire; (2) content design based on national and international clinical and educational standards; (3) application development using Visual Studio, ASP.NET framework with MVC architecture, and C#; and (4) usability assessment. The final evaluation involved 46 participants with HTN from the Hormoz Clinic, who used the application for one month, followed by an assessment using the Mobile Application Usability Questionnaire (MAUQ).

**Results:**

The HOPE application was designed with nine main tabs and 52 sub-tabs, covering key areas such as demographic information, comprehensive education, nutrition tracking, BP recording, medication management, and a dialogue panel for communication with the doctor. The overall usability evaluation for the application yielded an average score of 4.32 (on a 5-point Likert scale), which was categorized as a “very good” level. The highest average score (4.37) was assigned to the “User Interface and Satisfaction” dimension. A significant relationship was determined between satisfaction with the user interface and the participants’ level of education (P > 0.05).

**Conclusion:**

The HOPE demonstrated very good usability across all evaluated dimensions—ease of use, interface quality, and usefulness. The strong usability performance suggests that the application is well-designed and has high potential to effectively enhance self-care practices and could be a valuable tool in digital health management programs for patients with HTN. Future research should explore the long-term impacts of using HOPE on clinical outcomes and patient adherence, as well as its integration into routine healthcare practice to optimize HTN management.

## Introduction

Hypertension (HTN) is a chronic medical condition characterized by persistent elevated Blood Pressure (BP) levels in the arterial vessels [1]. Often asymptomatic in its early stages, HTN is the most prevalent chronic condition managed in primary care and is the foremost modifiable risk factor for cardiovascular disease (CVD) morbidity and mortality worldwide [2]. BP is the most significant risk factor for the global burden of CVD. Substantial evidence demonstrates that effective BP reduction significantly lowers the global burden of CVD. Despite this established knowledge, the prevalence of HTN continues to rise globally, underscoring an urgent need for health systems to prioritize and enhance strategies for its effective control and treatment [3].

A comprehensive global analysis published in 2021examined the epidemiological trends of HTN worldwide. This large-scale study, which brought together researchers from numerous countries (including 85 experts, one from Iran) synthesized data from 1,173 population-based studies conducted in 185 countries, encompassing a combined sample of more than 104 million participants [4].The analysis revealed that approximately 1.2 billion adults globally are affected by HTN. Moreover, HTN was identified as a major contributor to the global burden of disease, accounting for nearly 10 million deaths and 200 million cases of disability each year [5]. HTN is more prevalent in obese individuals, those with CVD, diabetics, smokers, less educated people, and rural residents [6].

HTN is highly prevalent in Iran, affecting approximately one-quarter of the adult population, with the burden disproportionately concentrated in adults aged ≥60 years [7,8]. Hormozgan Province is located in southern Iran, and its capital is Bandar Abbas. The population of Hormozgan province and Bandar Abbas city is also susceptible to HTN due to the consumption of local produced foods with high salt content. The prevalence of HTN in Bandar Abbas was reported to be between 5.4–19.4% of people over 30 years old [9]. In light of the high prevalence of HTN in Iran and Hormozgan, it is imperative for the Iranian healthcare system to devise novel strategies for the prevention, diagnosis, and management of CVDs [10]. One of these strategies is the use of Mobile health (mHealth) platforms, which, with an efficient, effective, and patient-centered approach, help address important gaps in BP management, including access to care and patient participation [11].

mHealth, a subset of telehealth, utilizes mobile devices to deliver healthcare [12]. This facilitates convenient access to healthcare providers for patients, enables more effective management of illnesses, and ultimately contributes to improved health status [13]. The prevailing utilization of mHealth entails the dissemination of preventative healthcare education among patients [14]. Additionally, mHealth has been employed for disease surveillance, treatment support, epidemic tracking, and chronic disease management [15].

The advent of advanced technologies, such as Bluetooth and motion detection sensors (e.g., accelerometer, gyroscope), has given rise to numerous applications tailored for chronic diseases, including diabetes [16], heart disease [17], kidney problems [18], and notably, High BP [19]. These technological advancements have facilitated enhanced access to medical services for patients with chronic conditions in comparison to the pre-technological era [20].

In the contemporary era, mHealth has emerged as a pragmatic and compelling alternative for enhancing HTN management [21]. This development offers significant benefits, including the potential for significant cost and time savings for patients, caregivers, and healthcare providers [22]. mHealth supports HTN management by enabling continuous BP monitoring, optimizing medication adherence, and promoting lifestyle modifications, including nutritional guidance [23]. This integrated approach addresses the multifactorial nature of HTN to improve disease control and prevent progression [24].

The issue of self-care as a personal care system for monitoring human health must be prioritized [14]. The adoption of mHealth for self-care enables individuals to proactively and deliberately regulate their activities, thereby fostering physical, mental, and social well-being, and ensuring a high quality of life [25]. While evidence supports the efficacy of mHealth apps for improving self-care behaviors and clinical outcomes in HTN, their successful implementation relies heavily on usability and contextual adaptation. High-quality apps that are both evidence-based and user-centered hold significant potential to bridge self-management gaps, especially in regions with high disease burden and limited digital health resources [26,27]

Self-care encompasses a range of aspects, including lifestyle modifications, health maintenance and enhancement, disease prevention, self-assessment, and the implementation of action to address treatment and rehabilitation needs [28]. This strategy has been resulted in a number of notable benefits, including a significant extension in lifespan, an enhancement in overall life satisfaction, and a concurrent reduction in the demand for medical consultations and referrals to medical centers [29].

A multitude of studies have documented the outcomes of mHealth applications in HTN self-care. These studies have indicated that the community, medical professionals, and patients recognize the capacity of mHealth to address the challenges associated with this condition [24]. Despite the contemporary growth in mHealth implementation in Iran, there is a paucity of research examining the current state and future trajectory of mHealth application in HBP management [30].

A study of mobile applications utilized in Iran revealed the absence of an official and stablished frame work for the design and scientific content of these applications hinders widespread adoption. The majority of these applications function within the domain of general information and routine services [20]. Our investigation has revealed that existing Iranian apps for HTN management lack scientific validation, standardized guideline-based design, and regulatory oversight, with limited functionality and integration—indicating a pressing need for validated, evidence-based, and well-designed mHealth tools like HOPE. These applications prioritize managing diet and physical activity to regulate BP. The objective is to maintain optimal weight and body composition and thereby mitigate the risk of HTN [20]. This underscores the necessity of apps designed to support comprehensive HTN self-care. Consequently, this study was conducted to design and evaluate a HTN self-care application to enable patients to access health services outside of clinical settings and manage their HTN.

## Materials and methods

The present study was conducted in four steps:

1. The determination of the data elements and functional requirements of the application is imperative for the effective design and implementation of the system.
2. The design of the application’s content is determined.
3. The development of the application is underway.
4. The present study will assess the usability of the given subject.

This study was approved by the Ethics Committee of the Deputy of Research and Technology of Hormozgan University of Medical Sciences (No: IR.HUMS.REC.1403.255). The details can be accessed at: https://ethics.research.ac.ir/EthicsProposalView.php?id=561062

### Step 1: Determination of the data elements and functional requirements of HOPE

At this stage, the research community included databases, previous reliable studies, digital library resources, cardiologists, and patients with HTN in Bandar Abbas city. A systematic search of databases and digital library resources was initiated to identify relevant studies. This approach was undertaken to extract the requirements of the application.

To gather scientific evidence related to the determination of data elements, the formulation of functional requirements a systematic and structured search of the scientific literature was conducted. This strategy adhered to the principles of systematic reviews to ensure comprehensiveness and process reproducibility.

A) Databases Searched:

The search was performed across four main electronic databases—PubMed, Scopus, Web of Science and Cochrane —to ensure broad coverage of the literature relevant to the study’s subject matter.

B) Search Terms:

Search terms were constructed by combining controlled vocabulary, such as MeSH terms, across three primary concepts: (1) The mHealth, (2) HTN (3) self-care

The Boolean operator AND was used to link the three core concepts, while the operator OR was employed to link synonyms within each concept.

C) Screening Method:

All retrieved records were imported into the reference management application EndNote and the screening process was executed in three stages following the PRISMA (Preferred Reporting Items for Systematic Reviews and Meta-Analyses) guidelines.

1. De-duplication: Initially, exact duplicate records were systematically removed.
2. Title and Abstract Screening: The remaining titles and abstracts were evaluated by two independent researchers, against the predefined initial inclusion criteria (e.g., relevance to the design/evaluation of similar applications, study type, and language of publication).
3. Full-Text Review: Full texts of articles deemed potentially eligible in the previous stage were retrieved and re-examined by the two independent researchers and the specific requirement for data extraction (functional requirements).
4. Ultimately, the data elements and system requirements were systematically extracted from the final set of included articles to serve as scientific evidence in step 1.

The 92 extracted data elements were categorized into seven primary domains. A structured questionnaire was subsequently developed, utilizing a five-point Likert scale ranging from “strongly agree (5)” to “strongly disagree (1),” to solicit input from both cardiologists and patients regarding the inclusion of each element in the application. This instrument was designed for self-administration and was completed independently by participants from both groups (See S1).

Any data elements that received an average score of 3.75 or more was approved, and those with an average score lower than that were excluded from the study. Prior to the completion of the questionnaire, verbal consent was obtained from patients. At this stage of the study, a total of 75 individuals were considered for inclusion in the study sample. This sample included 25 physicians and 50 patients. Subsequently, the collected data were subjected to analysis and review using SPSS26 application, which entailed the calculation of the mean score and standard deviation. The content of the questionnaire was validated using a Delphi method with an expert panel. The panel consisted of three cardiologists and three specialists in the domain of health information technology. The process was conducted in two rounds, with member feedback collected via a 5-point Likert scale in each round. A consensus threshold of >75% agreement served as the final criterion for item validation. The reliability of the questionnaire in the present study was also calculated and approved using the Cronbach’s alpha test, which yielded a coefficient of 0.91

### Step 2: HOPE content determination

Following a thorough review of the questionnaire, an expert panel was convened comprising a cardiologist, a health information technology (HIT) specialist, and a application designer. The panel’s objective was to identify the core features and content requirements for HOPE. The primary output of this panel was the development of an initial content architecture and layout for the application.

This collaborative process resulted in the design of the program. It is imperative to emphasize that the quality of content constitutes one of the most crucial elements in the realm of reliable health applications. To ensure the caliber of health information disseminated through the mobile application, the clinical and educational information requisite for the self-care application was obtained from national and international standards. The most salient of these standards or guidelines pertain to issues published by the World Health Organization [31], the American College of Cardiology (ACC) and American Heart Association (AHA) [32] and the Ministry of Health of Iran [33]These guidelines encompass a wide range of subjects, including physical activity, nutrition, lifestyle, high-risk behaviors, safety, and more. The information extracted from these protocols was utilized in the design of HOPE, particularly the education, BP, and medication modules, following validation by physicians and patients.

### Step 3: HOPE design

The outcome of this phase was the development of a functional, web-based application. HOPE architecture employed a client-server model to establish a secure platform accessible through standard web browsers. During this stage, operational requirements were formalized, and corresponding use case scenarios and system diagrams were produced. To design the mobile application user interface, the structural relationships and navigation flow between pages were wireframed using Figma application. Subsequently, an initial high-fidelity prototype of the smartphone application was developed by a collaborative team of user experience designers and application engineers. This prototype was directly informed by the previously approved feature specifications and system requirements.

The outcome of this stage was the development of a functional, web-based application. In this stage, operational requirements were extracted and scenarios and diagrams used were drawn. In order to design the mobile application user interface, the existing pages and their relationship with each other were modeled in Figma application. The initial model of the smartphone application was developed by design professionals and application development experts, who based their work on the approved features and requirements.

The application has been developed using C#, an object-oriented programming language designed by Microsoft as part of the  .NET platform. The language’s simplicity and robust capabilities have led to its extensive utilization in the realm of application development, particularly in the domains of applications, web development, and video games.

To address security, privacy, and data storage requirements, the system implements a multi-layered defense strategy. This includes employing HTTPS/TLS protocols to ensure encrypted data transmission, establishing robust user authentication and session management controls, and utilizing encrypted databases with strict, role-based access controls on secured servers for data storage at rest.

The prototype was ultimately subjected to a comprehensive review by the 25 experts during in-person validation sessions. This review was conducted to assess the program’s functionality, usability, and clinical appropriateness. The intervention delivers educational content through a multimedia format, including instructional videos, illustrative graphics, and supporting text. HOPE was developed as a web-based application to ensure universal accessibility and cross-platform compatibility. This architecture allows the program to operate consistently across all major operating systems via a standard web browser, thereby eliminating the need for platform-specific development and reducing long-term maintenance burdens.

### Step 4: The usability evaluation

At this stage, HOPE was made available to patients with HTN. Patients were selected at the Hormoz Clinic subsequent to receiving explanations regarding the objective and instructions for participating in the study, with particular emphasis placed on the confidentiality of the patients’ identities. Inclusion criteria comprised: 1) a clinical diagnosis of HTN (HTN), evidenced by at least three documented BP readings >140/90 mmHg; 2) age ≥ 18 years (To avoid ethical issues related to the presence of minors in the study); 3) ownership of a smartphone to access the web-based HOPE application; and 4) either personal or cohabitant literacy sufficient for application use. Individuals were excluded if they had a history of myocardial infarction, angiography, liver disease, or severe HTN. Severe HTN was defined as a sudden and marked elevation in BP, typically exceeding 180/120 mmHg.

The start date for recruitment of human participants was June 9, 2025, and the end date for the study observation period was July 19, 2025. convenience Sampling method was used. Participants voluntarily participated in the study, and informed consent was given verbally to the researcher for each participant.

To ensure participants’ understanding and voluntary agreement, a standardized verbal consent process was implemented. Each participant was asked a series of structured questions to confirm comprehension of the study’s purpose, procedures, and their rights as research subjects. Specifically, participants affirmed verbally that they: (1) understood the purpose of the study, (2) understood what their participation would involve, (3) acknowledged that participation was completely voluntary and that they could withdraw at any time without any consequences, and (4) agreed to participate based on the information provided. To create an auditable record of this consent, the entire verbal exchange was audio-recorded with the participant’s explicit prior permission. The responses of the participants were collected verbally.

This standardized procedure, confirming voluntary and informed participation, was reviewed and approved by the Institutional Review Board (IRB) as part of the ethical protocol ^for the HTN^ care program, ensuring full compliance with ethical standards for research involving human participants.

The app’s utilization and the study’s participation were promoted through announcements on the Hormoz Clinic bulletin boards. Ultimately, 52 individuals met the inclusion and exclusion criteria and were enrolled in the study.

At the beginning of the study period, participants completed a self-administered demographic questionnaire consisting of five items: age, gender, marital status, educational attainment, and employment status. In order to address the challenge of participant attrition, a WhatsApp group comprising these individuals was established. In this group, the utilization of the program was instructed through the medium of seven videos, with a duration of less than two minutes and a size of less than five megabytes. Furthermore, the utilization of the application was promoted through the dissemination of motivational messages and the administration of questions. Subsequently, the application link was disseminated via the WhatsApp group, and within a one-week period, patients were granted the opportunity to create an account in HOPE. In the event of technical difficulties, patients are advised to contact the application support team, who will provide guidance via message or telephone. A total of 52 accounts were created in the app, and users commenced a 40-day self-care period.

Forty days later, the usability questionnaire was given to the users, and HOPE’s usability was evaluated using the MAUQ tool (See S2). This tool was developed by Zho et al. in 2019. It contains two questionnaires. One is specific to apps used only by patients, and the other is for apps used by medical staff and patients. The first questionnaire was used in this study. This questionnaire has 18 questions in three categories. These dimensions are ease of use (five questions), Interface and satisfaction (seven questions), and usefulness (six questions). Users answered the questions of this self-administer questionnaire based on a 5-point Likert scale ranging from “strongly agree” (5) to “strongly disagree” (1). The total usability score and its cut points (e.g., excellent>70, 50_satisfactory_70, or poor<50) were determined [34]. The questionnaire was translated into Persian, and the eight-member expert panel, consisting of three cardiologists, two application designers, and three health information technology specialists, reviewed and approved its validity. The reliability of the questionnaire was calculated and confirmed by the Cronbach’s alpha test, which yielded a coefficient of 0.96. SPSS 26 application was used to analyze the data and calculate the mean and standard deviation. A chi-square test examined the relationship between demographic variables and usability.

## Results

### Findings related to determining HOPE requirements

Based on the study’s primary objective, a questionnaire was developed that included the data elements and capabilities required for the HTN self-care application (Table 1). According to the results, the data elements “height,” “skin color,” and “place of birth” received an average score of less than 3.75 from cardiologists and patients combined. Consequently, these data elements were deemed unnecessary.

**Table 1:** Average opinions of doctors and patients in the HTN application requirements questionnaire.

row	Class	row	Module	Average^*^		
Patients	Physician	Total
1	Demographic information	1	Age	28/4	5	52/4
		2	Sex	38/3	88/4	88/3
		3	Height	98/2	56/4	51/3
		4	Weight	10/4	92/4	37/4
		5	Family history of hypertension	44/4	84/4	57/4
		6	Skin color	38/2	04/4	92/2
		7	Place of birth	96/2	92/3	28/3
2	Education	8	Definition of hypertension	28/4	60/4	39/4
		9	Prevention of hypertension	42/4	72/4	52/4
		10	Causes of hypertension	51/4	68/4	57/4
		11	Complications of hypertension	51/4	76/4	59/4
		12	Predisposing factors	31/4	80/4	47/4
		13	Diagnosis of hypertension	50/4	71/4	57/4
		14	Measuring hypertension	45/4	78/4	56/4
		15	Importance of heredity in the disease	28/4	48/4	34/4
		16	Relationship of hypertension with increasing age	24/4	70/4	38/4
		17	Relationship of hypertension with the immune system	40/4	04/4	29/4
		18	Relationship of hypertension and diabetes	44/4	91/4	59/4
		19	Relationship of hypertension and kidney failure	44/4	91/4	59/4
		20	Relationship of hypertension with heart attack	44/4	96/4	60/4
		21	Relationship of hypertension with obesity	40/4	87/4	55/4
		22	Importance of nutrition in controlling hypertension	64/4	91/4	73/4
		23	Suitable food for rapid reduction of hypertension	58/4	22/4	47/4
		24	Suitable fruits for controlling BP	66/4	39/4	58/4
		25	Suitable vegetables for controlling hypertension	70/4	35/4	59/4
		26	Harmful fruits for hypertension	46/4	61/4	51/4
		27	Harmful vegetables for hypertension	46/4	61/4	51/4
		28	Suitable spices for controlling hypertension	56/4	13/4	42/4
		29	Relationship of hypertension with fast food	46/4	57/4	49/4
		30	Harmful foods for patients	72/4	87/4	77/4
		31	Suitable medicinal herbs for controlling	56/4	87/4	34/4
		32	Routine medications for controlling hypertension	54/4	65/4	58/4
		33	Use of text content	70/4	89/4	79/4
		34	Use of images	44/4	90/4	67/4
		35	Use of educational clips	63/4	78/4	70/4
		36	Search capability in the education tab	46/4	61/4	51/4
3	Nutrition	37	Record food intake	20/4	22/4	21/4
		38	Calculate calorie intake	20/4	22/4	21/4
		39	View calorie intake in a graph	35/4	54/4	44/4
		40	Control eating habits with a periodic questionnaire	56/4	20/4	38/4
		41	Record daily water intake (glasses)	44/4	80/3	23/4
		42	View water intake in a graph	20/4	22/4	21/4
4	Nutrition	43	Registering medications, you are taking	60/4	69/4	72/4
		44	Registering metoprolol	33/4	72/4	52/4
		45	Registering bisoprolol	52/4	87/4	65/4
		46	Registering carvedilol	62/4	62/4	62/4
		47	Registering captopril	31/4	90/4	60/4
		48	Registering enalapril	55/4	42/4	48/4
		49	Registering losartan	78/4	98/4	88/4
		50	Registering valsartan	76/4	85/4	80/4
		51	Registering amlodipine	63/4	65/4	64/4
		52	Registering hydrochlorothiazide	46/4	74/4	60/4
		53	Registering indapamide	25/4	81/4	53/4
		54	Registering spironolactone	65/4	83/4	74/4
		55	Registering eplerenone	41/4	68/4	54/4
		56	Registering metolazone	38/4	49/4	43/4
		57	Registering furosemide	56/4	68/4	60/4
		58	Registering telmisartan	76/4	85/4	80/4
		59	Registering irbesartan	78/3	42/4	10/4
		60	Registering atenolol	38/4	59/4	48/4
		61	Registering lisinopril	42/4	57/4	49/4
		62	Registering metoprolol	15/4	68/4	41/4
		63	Registering medications, you were previously taking	40/4	64/4	48/4
		64	Registering drug allergies	56/4	68/4	60/4
		65	Drug-drug interaction warning	56/4	44/4	52/4
		66	Warning of interference with painkillers	76/4	85/4	80/4
		67	Warning of interference with pregnancy	78/4	98/4	88/4
		68	Warning of interference with breastfeeding	31/4	90/4	60/4
		69	Warning of interference with tobacco use	51/4	72/4	61/4
		70	Warning of interference with alcohol use	42/4	68/4	55/4
		71	Warning of dietary interventions	77/4	85/4	81/4
		72	Reminder of taking high BP medications	62/4	80/4	68/4
5	Underlying diseases	73	Record history of treated diseases	40/4	71/4	50/4
		74	Record active hereditary diseases	36/4	17/4	30/4
		75	Record history of treated diseases of family members (father, mother, siblings)	22/4	4	15/4
		76	Record active diseases of family members (father, mother, siblings)	18/4	08/4	15/4
		77	Record active underlying diseases	36/4	63/4	45/4
		78	Record daily BP	69/4	80/4	74/4
		79	View trend of BP changes with graph	4	76/4	38/4
6	Exercise	80	Slow walking record	39/4	64/4	51/4
		81	Moderate walking record	42/4	63/4	52/4
		82	Fast walking record	44/4	58/4	51/4
		83	Calculate calories burned	80/4	74/4	77/4
7	Champion Physician	84	Send a message to the champion physician	68/4	44/4	60/4
		85	Receive a message from the champion physician	74/4	44/4	64/4
		86	View patient information by the champion physician if the patient allows	56/4	52/4	55/4
		87	View patient demographic information	41/4	73/4	57/4
		88	View BP changes	82/4	78/4	80/4
		89	View patient calorie intake	35/4	65/4	50/4
		90	View patient calorie consumption	41/4	62/4	51/4
		91	View patient physical activity	48/4	58/4	53/4
		92	View patient medication list	69/4	78/4	73/4

*The average of the total responses of patients and doctors and their sum for each question rated from 1 to 5.

### Findings related to determining HOPE content

Following a review of the requirements identified in the prior phase, alongside relevant clinical guidelines and protocols, the functional capabilities of the HOPE were delineated. These capabilities were subsequently structured into 9 tabs and 52 sub-tabs (Table 2)

**Table 2:** Main tabs and sub-tabs of HOPE.

	Tabs and subtabs		Details
1	Demographic information		age, gender, weight, family history of HTN
2	Education		includes an education tab (26 items) and a medication tab (18 items), where the education tab includes photos, text, and educational clips. The medication tab also introduces routine medications for controlling HTN and possible side effects of the medication.
3	Nutrition	Foods consumed	name, food calories and date and calorie intake chart by date
		Number of glasses of water consumed daily	–
		Nutritional Behavior Questionnaire	This questionnaire has 11 questions (see S3) and is used to promote proper nutritional behavior in people with HTN. The answers were expressed according to a five-point Likert scale (from most of the time (5) to never (1)). The last three questions were reverse scored. Finally, the application, based on the total score (11–55), shows the following results to the user: poor adherence level (11–22), moderate adherence level (23–33), good adherence level (34–44) and excellent adherence level (45–55).
4	BP		recording systolic and diastolic BP, line graph based on date
5	Medications	Recording of medications	
		Drug allergies	
		Medication reminder	
6	Underlying diseases	Diseases (current and past)	The user can record his other diseases, whether currently being treated or treated in the past, in the application
		Family history	
7	Exercise		(Daily walking rate in minutes and speed (slow, medium, fast)), calculation of calories burned in a table format based on date (
8	Profile		identity information (name and surname, contact number and address). Also, if the user wants the doctor to view the information recorded in the application, he can activate the “Allow access to the doctor to view information” option.
9	Dialogue panel		possibility of direct communication with the doctor supporting the application.

### Findings related to designing HOPE

**Fig 1:**
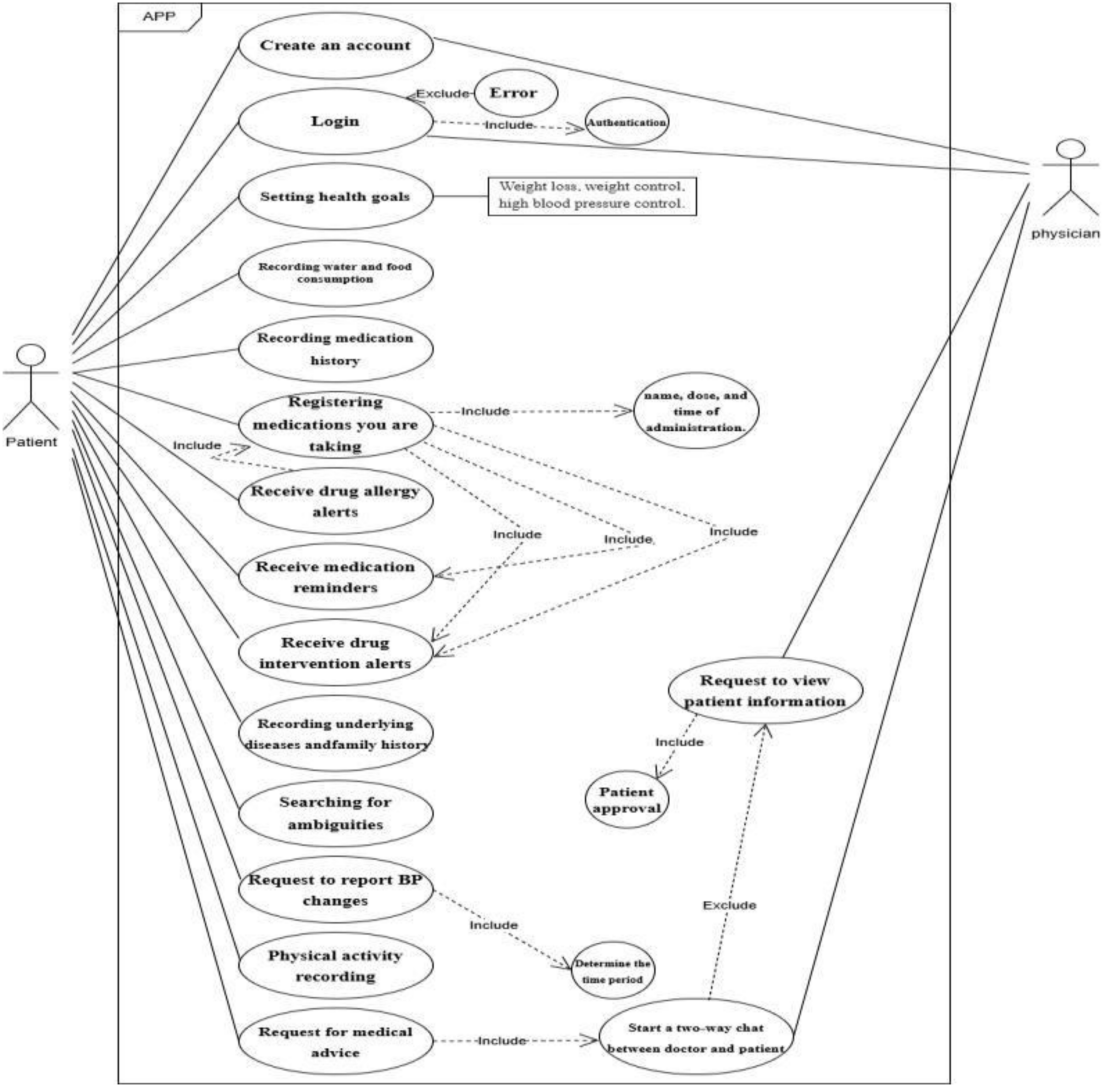
Use case diagram of the hypertension self‑care application. The diagram illustrates interactions between the user and the main functional components of the HOPE application.

A scenario of the stages of accessing and using applications from the user’s perspective, as well as the system’s response to user requests, was designed and arranged in Table 3. Then, using Draw.io, a diagram of the application (Fig 1) was designed based on the scenario. It was then made available to programming experts.

**Table 3:** A scenario of user requests and system responses to them.

Row	Scenario Description	User Request	System Response
1	Install and register	After downloading the application, the user easily registers using their email or phone number.	The application authenticates by sending a verification code to the phone number or email.
2	Create a profile	The user enters personal information including age, gender, weight, and medical history.	The application creates a user account based on the individual’s information.
3	Set health goals	The user can set their health goals; such as weight loss, weight control, high BP control.	The application provides appropriate programs and relevant advice based on the information entered.
4	Record water and food intake	The user can record their daily activities; type and amount of food consumed.	Using databases, the application provides analysis on user habits and suggests improvement tips.
5	Record medication history	The user can enter the names of medications they are already taking	The application checks medication history with current medications.
6	Record medications you are taking	The user can enter the names of medications they are currently taking	The application alerts the user if any drug interactions or matches with allergenic drugs are observed.
7	Receive reminders	The user enters the hours of medication use and the times of in-person appointments into the application.	The application sends reminders to the user to take medications, have periodic tests, and see a doctor.
8	Record underlying diseases and family history	The user can enter underlying and common diseases of themselves and their family.	The application provides the user with the necessary recommendations and training to prevent and control hereditary diseases.
9	Search and find ambiguities	The user can search for any ambiguity or question about their illness or ways to control, prevent, and care in the form of a sentence or keyword.	The application uses a database to provide analysis on user searches and suggests improvement tips to the user using the training tab.
10	Periodic reporting	The user registers a reporting request by specifying the time period and type of report.	The application periodically provides a report on the user’s health status; this report includes changes in weight, physical activity level, and progress towards set goals.
11	Record physical activities	The user enters the method and amount of their daily walking.	The application shows the user the number of calories burned based on a graph with the ability to set the time interval.
12	Receive medical advice	Users can request advice from a support doctor.	If needed, the user can consult with specialist doctors via chat or video call.

**Fig 2:**
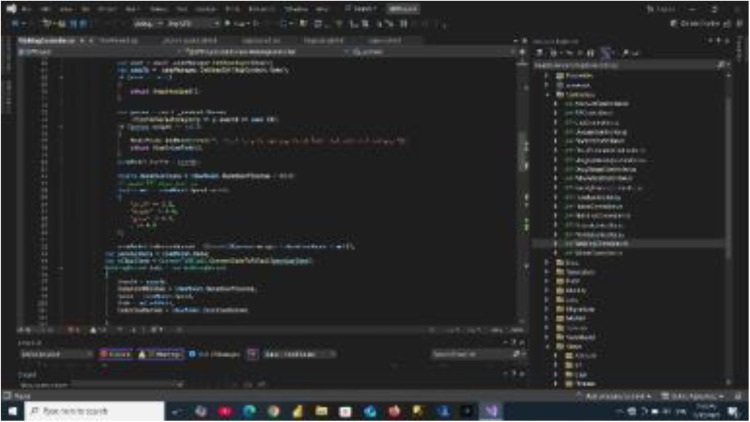
Coding architecture of the hypertension self‑care application. Shows the development framework and programming structure used to implement the HOPE application using ASP.NET MVC and C#.

**Fig 3:**
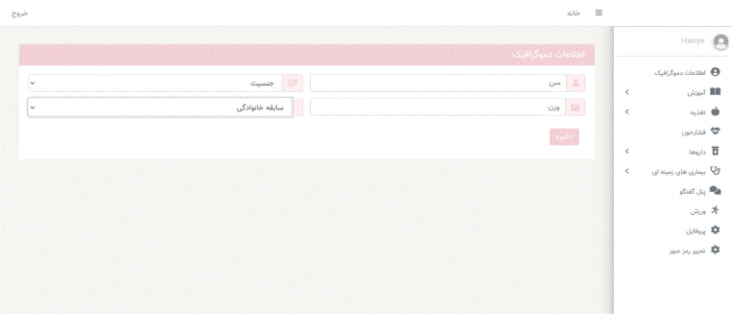
Demographic information tab of the HOPE application. Displays user demographic details and personal health information required for system personalization.

**Fig 4:**
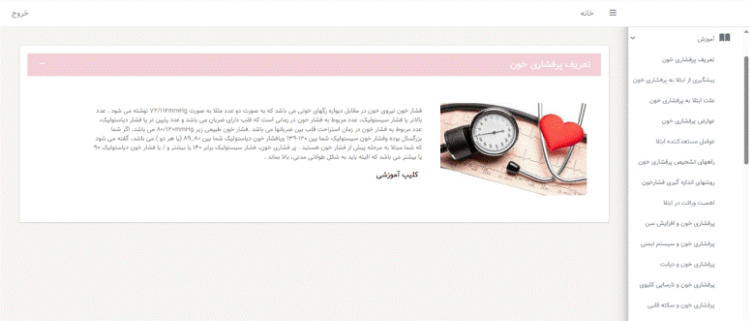
Education tab of the HOPE application. Provides educational materials and guidance to support hypertension self‑care and disease management.

**Fig 5:**
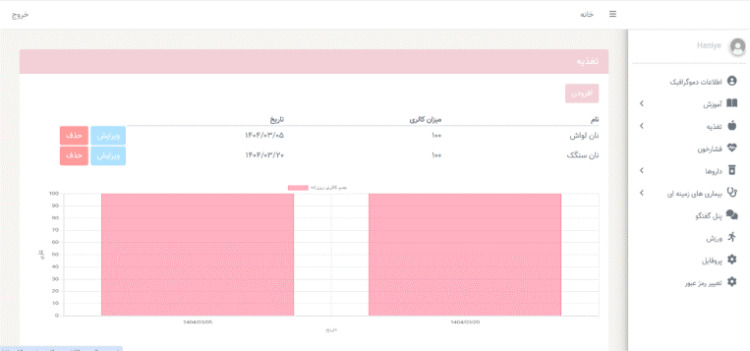
Nutrition tab of the HOPE application. Presents dietary recommendations and nutrition guidance for patients with hypertension.

**Fig 6:**
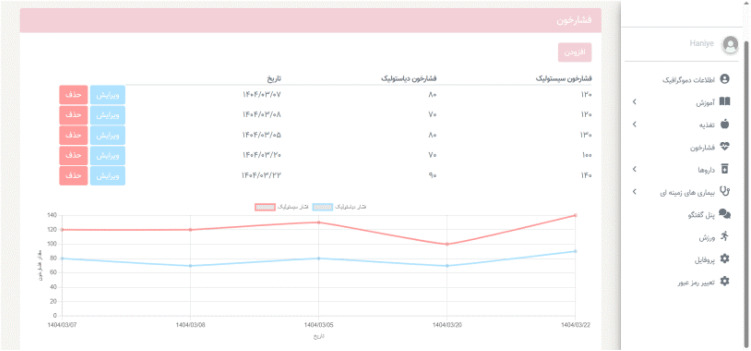
Blood pressure monitoring tab. Allows users to record and track their blood pressure measurements over time.

**Fig 7:**
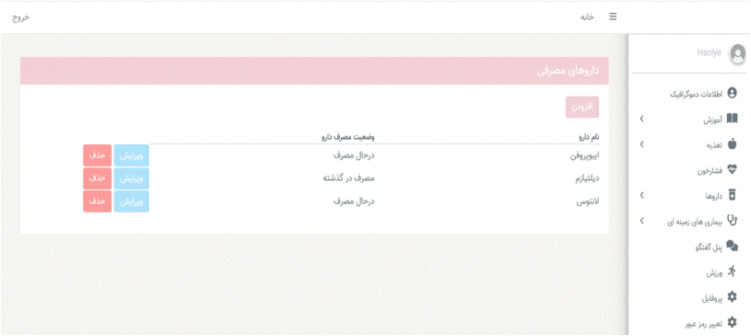
Medications management tab. Enables users to record prescribed medications and follow treatment schedules.

**Fig 8:**
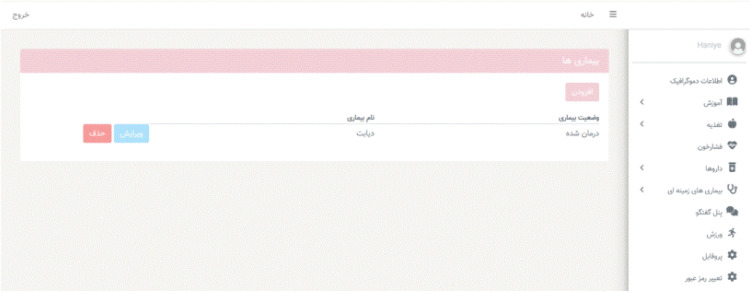
Underlying diseases tab. Displays information related to comorbid conditions associated with hypertension.

**Fig 9:**
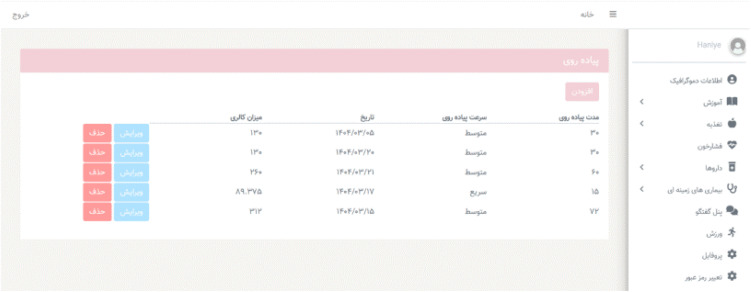
Physical exercises tab. Provides recommended exercises and physical activity guidance for hypertension management.

**Fig 10:**
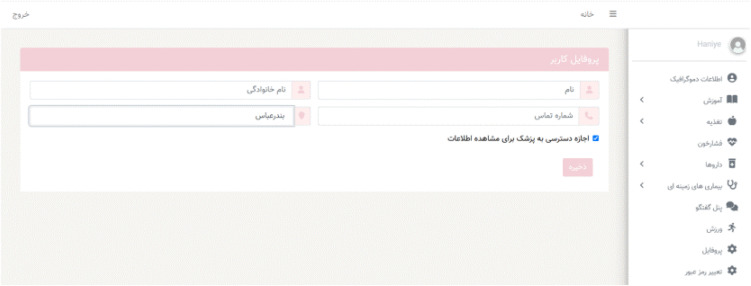
User profile tab of the HOPE application. Displays personal account settings and user profile information.

**Fig 11:**
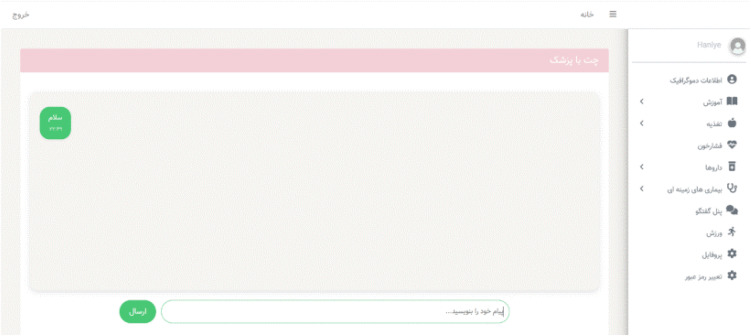
Dialogue panel tab of the HOPE application. This tab enables communication between the user and the system for guidance, questions, and interactive support related to hypertension self‑care.

After a face-to-face meeting to review the design requirements and the conceptual model of the designed application, coding began. During programming, communication with the programmer was carried out in person (three weekly sessions), by telephone, and virtually (in cases where necessary, via WhatsApp) in order to resolve ambiguities and make necessary corrections before the final review. Coding was carried out according to Fig 2 using Visual Studio application, the ASP.NET framework with MVC architecture, and the C# programming language. Also, screenshots of the nine main tabs are provided in Figs 3–11, including the demographic information tab (Fig 3), education tab (Fig 4), nutrition tab (Fig 5), blood pressure monitoring tab (Fig 6), medication management tab (Fig 7), underlying diseases tab (Fig 8), physical exercises tab (Fig 9), user profile tab (Fig 10), and dialogue panel tab (Fig 11).

### Findings related to HOPE usability assessment

Of the 52 initial participants who engaged with the application over the 40-day intervention period, 46 (88.5%) completed the final questionnaire in its entirety, while six (11.5%) were lost to follow-up. Reasons for attrition included: surgical intervention (n = 2), loss of contact (n = 2), and failure to record any data (n = 2). Their educational levels ranged from illiteracy to a Ph.D., with cycle and diploma degrees being the most common (Table 4). The response rate to the usability questionnaire by the participants at the end of the intervention period is also presented in Table 5.

**Table 4:** Demographic variables and relationship with usability dimensions.

Usability dimensions			Demographics		N (%)
Ease of use	Interface and satisfaction	Usefulness			
0.2	0.06	0.6	Sex	Male	20 (43.5)
				Female	26 (56.5)
0.7	0.6	0.4		Average age (year)	54.56
0.9	0.8	0.7	Marital Status	Single	3 (6.5)
				Married	40 (86.9)
				Other (deceased/ spouse)	3 (6.5)
0.7	0.02	0.2	Educational status (literacy level)	Illiterate	3 (6.5)
				Primary School	6 (13)
				Under diploma	6 (13)
				Diploma	16 (34.8)
				Associate Degree	4 (8.7)
				Bachelor Degree	6
				Master Degree	4 (8.7)
				PHD	1 (2.2)
0.7	0.5	0.1	Employment status	Housewife	15 (32.6)
				Student	1 (2.2)
				Worker	1 (2.2)
				Employee	12 (26.1)
				Retired	17 (36.9)

**Table 5:** Usability questionnaire response rate.

Row	Questions	Strongly Agree	Agree	Neutral	Disagree	Strongly Disagree
Ease of use (alpha = 0.886), 5 items (MAUQ_E)						
1	The app was easy to use.	23	21	1	0	0
2	It was easy for me to learn how to use this app	25	15	4	1	0
3	When moving between the screens of the application, the navigation was consistent.	20	14	10	1	0
4	The interface of this app gave me the possibility to use all provided features (such as entering information, response to reminders, viewing information)	24	17	3	1	0
5	Whenever I made a mistake in using the app, I could correct my mistake easily and quickly.	21	14	8	2	0
Interface and satisfaction (alpha = 0.927), 7 items						
6	I like the interface of the app	25	16	3	1	0
7	Information in the app was well organized; therefore, I could easily find information I needed	27	13	4	1	0
8	This app has verified and provided enough information for me to know the progress of my activity.	18	18	8	1	0
9	I feel comfortable using this app in public.	20	20	4	1	0
10	The time required to use this application was suitable for me.	27	12	5	1	0
11	I will use this app again	22	15	6	2	0
12	Overall, I am satisfied with this app.	29	12	3	1	0
Usefulness (alpha = 0.930), 6 items						
13	This app will be useful for my health and well-being.		17	2	1	0
14	This app improved my access to healthcare services.		15	7	1	0
15	This app helped me manage my health effectively.		15	7	1	0
16	This app has all the features and functions that I expected.		16	6	1	0
17	I could use this app even when the internet was weak or not connected.		8	15	4	0
18	This app provides an acceptable way to receive healthcare services such as accessing educational materials, tracking my activities, and performing self-assessments.		22	2	1	0

The application’s average usability score was 4.32. The highest average score among the usability criteria was assigned to the user Interface and satisfaction, with an average score of 4.37. The application’s ease of use and usefulness received average scores of 4.28 and 4.26, respectively. Since a score range of 3.75 is considered very good, the findings showed that all application usability dimensions, as well as the overall usability evaluation, were at very good levels.

It was also determined that there was a significant relationship between satisfaction with the user interface and the participants’ level of education (P > 0.02). No such relationships were found between any other usability dimensions and demographic variables (Table 4).

## Discussion

Considering the importance and impact of self-care application in BP management, as well as the research gap in Hormozgan, this study was conducted with the aim of designing and evaluating a HTN self-care application. The present study was performed in four steps determining requirements, examining program capabilities, designing and presenting a conceptual model, and finally evaluating usability. HOPE was designed in the form of 9 main tabs and 52 data elements, and its usability was reported to be (4.32/5), with particularly strong performance in user interface satisfaction.

These findings are significant within the Iranian digital health landscape, where a recognized scarcity of scientifically validated, guideline-based applications exists for chronic disease management [30]. More broadly, this work contributes to the global evidence base on the design and implementation of mHealth solutions in low- and middle-income country (LMIC) settings, where contextual adaptation is paramount for success.

Another study was performed in Iran for determining the information content and functions of an Application for Nutrition Management in Patients with Type 2 Diabetes [16]. To determine the information content and functions of the application, the researchers created a five-point Likert scale checklist consisting of six sections based on a review of clinical guidelines and specialized databases. Then, unlike the present study, they gave the checklist to only ten endocrinologists and metabolism, internal medicine, and nutrition specialists, and approved cases whose average score was higher than 3.75 [16].

In the third step, HOPE was designed based on physicians and patients’ inputs. This is a common that has long history in mHealth studies. For example, Toolaroud and his colleagues designed a Mobile app for Self-Management of sever burn injuries based on the feedback and comments obtained from the participants. They designed the requirements and operation of the program in the form of conceptual models (how to display the content of the program) using Edraw application. Then, its validity was evaluated by a focused group of experts. The steps of this study were similar to the steps selected by the researchers of the present study, but in the third stage, we used different tools to design and code the program [35].

HOPE’s comprehensive architecture—integrating education, monitoring, and patient-provider communication—sets it apart from many existing applications in Iran. By embedding content from WHO, ACC/AHA, and Iranian national guidelines, HOPE moves beyond generic tracking to deliver structured, credible self-management support. This approach mirrors the successful framework of apps like those evaluated by [36], which combined medication reminders with educational content to significantly improve adherence and BP control. The inclusion of a “Dialogue Panel” is a progressive feature, aligning with global shifts towards integrated telehealth. While its utility was not independently assessed here, similar communication functionalities have been linked to improved therapeutic alliance and patient empowerment in chronic care management [21].

In line with the final goal of the present study, the usability of HOPE was evaluated from the perspective of patients. The results of this study indicated that the self-care application for HTN demonstrated very good usability in three dimensions: ease of use, user interface and satisfaction and usefulness. The ease of use showed that users were able to benefit from the app’s features and perform required activities without complex training. This is an important advantage, as health applications should be accessible to a wide range of users with varying levels of health literacy. The well-organized and intuitive interface also contributed to a smooth user experience, reducing confusion and enhancing interaction between users and the system.

The “very good” usability rating across all dimensions (interface, ease of use, and usefulness) is a critical first-step validation. User satisfaction and usefulness play a crucial role in continuing usage and, consequently, in achieving behavioral changes in health management [37]. The usability results compare favorably with other usability evaluations of HTN apps in other studies. For instance, Alessa et al. (2019), in a review of commercially available apps, noted that poor usability and lack of evidence-based content were major barriers to effectiveness [19]. The systematic, multi-stakeholder requirement analysis (involving cardiologists and patients) employed in HOPE’s design likely mitigated these common pitfalls, resulting in an intuitive and relevant tool. However, a recent study by Salabi et al. demonstrated superior usability scores, attributable to two primary factors: the limited functional capabilities of the app and the elevated health literacy levels among its participants [38].

The highest score was in the “Interface and Satisfaction” dimension. This finding aligns with the principle that aesthetic appeal and intuitive navigation are key drivers of initial engagement in mHealth interventions [24,39]. Similarly, Alessa et al. (2019) found that a simple design and effective interaction between users and the app were critical to user engagement and long-term adoption [19]. However, it contrasts with some studies where “perceived usefulness” was the dominant factor for long-term adherence [23]. This discrepancy may reflect the novelty effect or indicate that for our cohort in the initial adoption phase, a positive first impression was paramount. Another reason could be the simple and understandable design of the user interface. cited challenges related to complex user interfaces or information overload created main challenges for users, specially novels [27]. User satisfaction and perceived usefulness play a crucial role in continuing usage and, consequently, in achieving behavioral changes in health management [40].

A salient finding was the significant relationship between interface satisfaction and participants’ education level. This underscores a persistent digital divide, even within populations using smartphones. Similar patterns have been observed in other LMIC mHealth studies, where higher educational attainment often correlates with greater digital comfort and more critical appraisal of app design [27]. This finding has important implementation implications: while HOPE was usable across the spectrum, scaling its impact will require complementary strategies, such as simplified digital literacy support or family-assisted use models, to ensure equitable access and benefit for less-educated populations, who are also at higher risk for poorly controlled HTN [41].

Overall, the alignment between this study and prior research supports the effectiveness of the design strategies implemented in the app and highlights the importance of focusing on the core aspects of usability for the success of electronic health interventions targeting patients with HTN.

This study, in common with prior research, has several limitations that warrant consideration. First, the findings are constrained by the study setting, as data collection was conducted at an outpatient center in southern Iran. Although the sample sizes for both the requirements determination and usability assessment phases exceed those reported in comparable studies, caution is advised when generalizing the results to broader national populations.

Second, the data collected during the requirements determination and usability evaluation phases relied on self-report measures from both physicians and patients. This methodological approach introduces the potential for self-report bias, which may influence the validity of the findings.

Third, the use of convenient sampling, while appropriate for the study’s exploratory aims, may have introduced selection bias, thereby limiting the representativeness of the participant pool.

Finally, the study population consisted exclusively of outpatients with HTN, who typically present with less severe clinical conditions compared to hospitalized patients. Consequently, the results should be interpreted with caution, as they may not be fully generalizable to inpatient or more acute hypertensive populations.

## Conclusion

This study demonstrates that human-centered design and iterative evaluations can yield effective, user-friendly digital tools for chronic disease management. HOPE exemplifies a model with potential for integration into e-health structures and for supporting active self-care in hypertensive patients. The findings indicated that HOPE demonstrated satisfactory usability due to its user-friendly design, coherent component organization, and scientifically valid content. Evaluation across ease of use, interface quality, and usefulness confirmed that it successfully met user expectations, enabling practical application without requiring complex training. Overall, the app provides a suitable platform for self-care and holds strong potential for integration into electronic health management systems.

Future research should expand its educational content, incorporate advanced interactive modules, and assess its long-term impact on clinical parameters. It is important to emphasize that a comprehensive assessment of the clinical implications of HOPE necessitates its long-term application (e.g., over a six-month period) to accurately evaluate its effects on pertinent clinical parameters and, ultimately, on BP management outcomes.

## Supporting information

S1 Hope Application Requirements Questionnaire. (DOCX)

S2 Mobile Application Usability Questionnaire. (DOCX)

S3 Nutrition Questionnaire. (DOCX)
