# Supplementary material for: Development and usability evaluation of HOPE: A patient-centered mHealth application for HTN self-management in Iran
Source: PLoS One. 2026 Jun 17;21(6):e0344541. doi: 10.1371/journal.pone.0344541 (PMC13274884; doi:10.1371/journal.pone.0344541)
Supplement: S1 — (DOCX) [file pone.0344541.s001.docx]

**Hope Application Requirements Questionnaire**

Dear participant, please indicate your level of agreement with each of the following options from 5 to 1 (from Strongly Agree to Strongly Disagree).

| row | Classification | row | Module | Strongly Agree | Agree | **Neutral** | Disagree | Strongly Disagree |
| --- | --- | --- | --- | --- | --- | --- | --- | --- |
| 1 | Demographic information | 1 | Age |  |  |  |  |  |
|  |  | 2 | Sex |  |  |  |  |  |
|  |  | 3 | Height |  |  |  |  |  |
|  |  | 4 | Weight |  |  |  |  |  |
|  |  | 5 | Family history of hypertension |  |  |  |  |  |
|  |  | 6 | Skin color |  |  |  |  |  |
|  |  | 7 | Place of birth |  |  |  |  |  |
| 2 | Education | 8 | Definition of hypertension |  |  |  |  |  |
|  |  | 9 | Prevention of hypertension |  |  |  |  |  |
|  |  | 10 | Causes of hypertension |  |  |  |  |  |
|  |  | 11 | Complications of hypertension |  |  |  |  |  |
|  |  | 12 | Predisposing factors |  |  |  |  |  |
|  |  | 13 | Diagnosis of hypertension |  |  |  |  |  |
|  |  | 14 | Measuring hypertension |  |  |  |  |  |
|  |  | 15 | Importance of heredity in the disease |  |  |  |  |  |
|  |  | 16 | Relationship of hypertension with increasing age |  |  |  |  |  |
|  |  | 17 | Relationship of hypertension with the immune system |  |  |  |  |  |
|  |  | 18 | Relationship of hypertension and diabetes |  |  |  |  |  |
|  |  | 19 | Relationship of hypertension and kidney failure |  |  |  |  |  |
|  |  | 20 | Relationship of hypertension with heart attack |  |  |  |  |  |
|  |  | 21 | Relationship of hypertension with obesity |  |  |  |  |  |
|  |  | 22 | Importance of nutrition in controlling hypertension |  |  |  |  |  |
|  |  | 23 | Suitable food for rapid reduction of hypertension |  |  |  |  |  |
|  |  | 24 | Suitable fruits for controlling blood pressure |  |  |  |  |  |
|  |  | 25 | Suitable vegetables for controlling hypertension |  |  |  |  |  |
|  |  | 26 | Harmful fruits for hypertension |  |  |  |  |  |
|  |  | 27 | Harmful vegetables for hypertension |  |  |  |  |  |
|  |  | 28 | Suitable spices for controlling hypertension |  |  |  |  |  |
|  |  | 29 | Relationship of hypertension with fast food |  |  |  |  |  |
|  |  | 30 | Harmful foods for patients |  |  |  |  |  |
|  |  | 31 | Suitable medicinal herbs for controlling |  |  |  |  |  |
|  |  | 32 | Routine medications for controlling hypertension |  |  |  |  |  |
|  |  | 33 | Use of text content |  |  |  |  |  |
|  |  | 34 | Use of images |  |  |  |  |  |
|  |  | 35 | Use of educational clips |  |  |  |  |  |
|  |  | 36 | Search capability in the education tab |  |  |  |  |  |
| 3 | Nutrition | 37 | Record food intake |  |  |  |  |  |
|  |  | 38 | Calculate calorie intake |  |  |  |  |  |
|  |  | 39 | View calorie intake in a graph |  |  |  |  |  |
|  |  | 40 | Control eating habits with a periodic questionnaire |  |  |  |  |  |
|  |  | 41 | Record daily water intake (glasses) |  |  |  |  |  |
|  |  | 42 | View water intake in a graph |  |  |  |  |  |
| 4 | Nutrition | 43 | Registering medications you are taking |  |  |  |  |  |
|  |  | 44 | Registering metoprolol |  |  |  |  |  |
|  |  | 45 | Registering bisoprolol |  |  |  |  |  |
|  |  | 46 | Registering carvedilol |  |  |  |  |  |
|  |  | 47 | Registering captopril |  |  |  |  |  |
|  |  | 48 | Registering enalapril |  |  |  |  |  |
|  |  | 49 | Registering losartan |  |  |  |  |  |
|  |  | 50 | Registering valsartan |  |  |  |  |  |
|  |  | 51 | Registering amlodipine |  |  |  |  |  |
|  |  | 52 | Registering hydrochlorothiazide |  |  |  |  |  |
|  |  | 53 | Registering indapamide |  |  |  |  |  |
|  |  | 54 | Registering spironolactone |  |  |  |  |  |
|  |  | 55 | Registering eplerenone |  |  |  |  |  |
|  |  | 56 | Registering metolazone |  |  |  |  |  |
|  |  | 57 | Registering furosemide |  |  |  |  |  |
|  |  | 58 | Registering telmisartan |  |  |  |  |  |
|  |  | 59 | Registering irbisartan |  |  |  |  |  |
|  |  | 60 | Registering atenolol |  |  |  |  |  |
|  |  | 61 | Registering lisinopril |  |  |  |  |  |
|  |  | 62 | Registering metoprolol |  |  |  |  |  |
|  |  | 63 | Registering medications you were previously taking |  |  |  |  |  |
|  |  | 64 | Registering drug allergies |  |  |  |  |  |
|  |  | 65 | Drug-drug interaction warning |  |  |  |  |  |
|  |  | 66 | Warning of interference with painkillers |  |  |  |  |  |
|  |  | 67 | Warning of interference with pregnancy |  |  |  |  |  |
|  |  | 68 | Warning of interference with breastfeeding |  |  |  |  |  |
|  |  | 69 | Warning of interference with tobacco use |  |  |  |  |  |
|  |  | 70 | Warning of interference with alcohol use |  |  |  |  |  |
|  |  | 71 | Warning of dietary interventions |  |  |  |  |  |
|  |  | 72 | Reminder of taking high blood pressure medications |  |  |  |  |  |
| 5 | Underlying diseases | 73 | Record history of treated diseases |  |  |  |  |  |
|  |  | 74 | Record active hereditary diseases |  |  |  |  |  |
|  |  | 75 | Record history of treated diseases of family members (father, mother, siblings) |  |  |  |  |  |
|  |  | 76 | Record active diseases of family members (father, mother, siblings) |  |  |  |  |  |
|  |  | 77 | Record active underlying diseases |  |  |  |  |  |
|  |  | 78 | Record daily blood pressure |  |  |  |  |  |
|  |  | 79 | View trend of blood pressure changes with graph |  |  |  |  |  |
| 6 | Exercise | 80 | Slow walking record |  |  |  |  |  |
|  |  | 81 | Moderate walking record |  |  |  |  |  |
|  |  | 82 | Fast walking record |  |  |  |  |  |
|  |  | 83 | Calculate calories burned |  |  |  |  |  |
| 7 | Supporting physician | 84 | Send a message to the supporting doctor |  |  |  |  |  |
|  |  | 85 | Receive a message from the supporting doctor |  |  |  |  |  |
|  |  | 86 | View patient information by the doctor if the patient allows |  |  |  |  |  |
|  |  | 87 | View patient demographic information |  |  |  |  |  |
|  |  | 88 | View blood pressure changes |  |  |  |  |  |
|  |  | 89 | View patient calorie intake |  |  |  |  |  |
|  |  | 90 | View patient calorie consumption |  |  |  |  |  |
|  |  | 91 | View patient physical activity |  |  |  |  |  |
|  |  | 92 | View patient medication list |  |  |  |  |  |
