# Supplementary material for: Development and usability evaluation of HOPE: A patient-centered mHealth application for HTN self-management in Iran
Source: PLoS One. 2026 Jun 17;21(6):e0344541. doi: 10.1371/journal.pone.0344541 (PMC13274884; doi:10.1371/journal.pone.0344541)
Supplement: S2 — (DOCX) [file pone.0344541.s002.docx]

| Row | Questions | Strongly Agree | Agree | **Neutral** | Disagree | Strongly Disagree |
| --- | --- | --- | --- | --- | --- | --- |
| 1 | The app was easy to use. |  |  |  |  |  |
| 2 | It was easy for me to learn how to use this app |  |  |  |  |  |
| 3 | When moving between the screens of the application, the navigation was consistent. |  |  |  |  |  |
| 4 | The interface of this app gave me the possibility to use all provided features (such as entering information, response to reminders,  viewing information) |  |  |  |  |  |
| 5 | Whenever I made a mistake in using the app, I could correct my mistake easily and quickly. |  |  |  |  |  |
| 6 | I like the interface of the app |  |  |  |  |  |
| 7 | Information in the app was well organized; therefore, I could easily find information I needed |  |  |  |  |  |
| 8 | This app has verified and provided enough information for me to know the progress of my activity. |  |  |  |  |  |
| 9 | I feel comfortable using this app in public. |  |  |  |  |  |
| 10 | The time required to use this application was suitable for me. |  |  |  |  |  |
| 11 | I will use this app again |  |  |  |  |  |
| 12 | Overall, I am satisfied with this app. |  |  |  |  |  |
| 13 | This app will be useful for my health and well-being. |  |  |  |  |  |
| 14 | This app improved my access to healthcare services. |  |  |  |  |  |
| 15 | This app helped me manage my health effectively. |  |  |  |  |  |
| 16 | This app has all the features and functions that I expected. |  |  |  |  |  |
| 17 | I could use this app even when the internet was weak or not connected. |  |  |  |  |  |
| 18 | This app provides an acceptable way to receive healthcare services such as accessing educational materials, tracking my activities,  and performing self-assessments. |  |  |  |  |  |

**Mobile Application Usability Questionnaire (MAUQ)**
