# Supplementary material for: Development and usability evaluation of HOPE: A patient-centered mHealth application for HTN self-management in Iran
Source: PLoS One. 2026 Jun 17;21(6):e0344541. doi: 10.1371/journal.pone.0344541 (PMC13274884; doi:10.1371/journal.pone.0344541)
Supplement: S3 — (DOCX) [file pone.0344541.s003.docx]

Nutrition Questionnaire

| Never | little | Sometimes | all the time | Most of the time | Feeding behavior |
| --- | --- | --- | --- | --- | --- |
|  |  |  |  |  | I consume at least 7 handfuls of bread and whole grains daily |
|  |  |  |  |  | I consume at least 4 cups of raw vegetables or 2 cups of cooked vegetables daily. |
|  |  |  |  |  | I eat at least 4 medium-sized pieces of fruit every day. |
|  |  |  |  |  | I consume at least 2 servings of low-fat dairy products (low-fat milk, low-fat yogurt, low-fat buttermilk, low-fat cheese) daily. |
|  |  |  |  |  | I consume less than 2 servings of chicken, fish, lean meat, and eggs per day. |
|  |  |  |  |  | I eat at least a quarter cup of nuts, seeds, and legumes daily (at least 2 cups per week). |
|  |  |  |  |  | I consume less than 2 servings of various vegetable oils and fats (low-fat mayonnaise) daily. |
|  |  |  |  |  | I consume sweets less than 5 times a week. |
|  |  |  |  |  | I have a habit of using a salt shaker at the table. |
|  |  |  |  |  | I have a habit of using canned foods and ready-to-eat frozen foods. |
|  |  |  |  |  | I have a habit of consuming various types of fast food and processed meats such as sausages. |
